# Rapid Detection of Recurrent Non-Muscle Invasive Bladder Cancer in Urine Using ATR-FTIR Technology

**DOI:** 10.3390/molecules27248890

**Published:** 2022-12-14

**Authors:** Abdullah I. El-Falouji, Dalia M. Sabri, Naira M. Lotfi, Doaa M. Medany, Samar A. Mohamed, Mai Alaa-eldin, Amr Mounir Selim, Asmaa A. El Leithy, Haitham Kalil, Ahmed El-Tobgy, Ahmed Mohamed

**Affiliations:** 1 Institute of Biotechnology for Postgraduate Studies and Research (IBPR), Suez Canal University, Ismailia 41522, Egypt; 2 Surgical Oncology Department, National Cancer Institute, Cairo University, Cairo 11796, Egypt; 3 College of Biotechnology, Misr University for Science and Technology, Giza 12562, Egypt; 4 Chemistry Department, Faculty of Science, Suez Canal University, Ismailia 41522, Egypt; 5 Chemistry Department, Cleveland State University, Cleveland, OH 44115, USA; 6 Department of Urology, Faculty of Medicine, Suez Canal University, Ismailia 41522, Egypt; 7 Division of Bioinformatics and Colonial Foundation Healthy Ageing Centre, Walter and Eliza Hall Institute of Medical Research, Melbourne, VIC 3052, Australia

**Keywords:** liquid biopsy, bladder cancer, FTIR, machine learning

## Abstract

Non-muscle Invasive Bladder Cancer (NMIBC) accounts for 80% of all bladder cancers. Although it is mostly low-grade tumors, its high recurrence rate necessitates three-times-monthly follow-ups and cystoscopy examinations to detect and prevent its progression. A rapid liquid biopsy-based assay is needed to improve detection and reduce complications from invasive cystoscopy. Here, we present a rapid spectroscopic method to detect the recurrence of NMIBC in urine. Urine samples from previously-diagnosed NMIBC patients (*n* = 62) were collected during their follow-up visits before cystoscopy examination. Cystoscopy results were recorded (41 cancer-free and 21 recurrence) and attenuated total refraction Fourier transform infrared (ATR-FTIR) spectra were acquired from urine samples using direct application. Spectral processing and normalization were optimized using parameter grid searching. We assessed their technical variability through multivariate analysis and principal component analysis (PCA). We assessed 35 machine learning models on a training set (70%), and the performance was evaluated on a held-out test set (30%). A Regularized Random Forests (RRF) model achieved a 0.92 area under the receiver operating characteristic (AUROC) with 86% sensitivity and 77% specificity. In conclusion, our spectroscopic liquid biopsy approach provides a promising technique for the early identification of NMIBC with a less invasive examination.

## 1. Introduction

Early, non-invasive, and conclusive disease identification relates to several favorable outcomes. These include reducing the strain on healthcare expenditures that are already stretched tight since the COVID-19 pandemic and employing therapeutic procedures that reduce morbidity and death at an early stage. There are indications that some routinely-employed clinical tests are unsuitable or misleading. In addition, they typically employ inadequate single illness markers when multiple factors are at play. The growing subject of metabolomics includes a technique known as metabolic fingerprinting, which is a form of high throughput and is the universal analysis used to differentiate samples swiftly and correctly. The differentiation is based on the change in a selective area as a result of different statuses of disease or changes in the biological environment. Using infrared or Raman spectroscopy, metabolic fingerprinting has been presented as a potential technique and a powerful tool for disease diagnostics [1].

Approximately 70–75% of urothelial carcinoma cases are classified as non-muscle-invasive bladder cancer (NMIBC). In NMIBC, the tumor is not invading the muscularis propria and is confined to mucosa and submucosa layers [2,3]. NMIBC is characterized by a high recurrence rate of 60–70% while, in 10–20% of patients, the tumor will develop a progression to muscle invasive carcinoma (MIBC).

Due to the high recurrence rate, a follow-up using a cystoscopy examination is required frequently according to European Association of Urology (EAU) Guidelines [4]. However, cystoscopy could give an indefinite diagnosis in patients with active inflammation, indwelling catheter, or abnormal appearance of the bladder mucosa. Besides, it is an invasive, painful, and uncomfortable procedure with the risk of causing urinary tract infections in 10% of the patients [5,6]. Therefore, a non-invasive liquid biopsy marker for bladder carcinoma diagnosis is needed.

There is great potential for using urine-based tumor markers as a non-invasive, affordable method for diagnosing and monitoring the progression of tumors, replacing the use of cystoscopy in tumor follow-up [7]. Urine samples have distinct advantages such as non-invasive sampling, and the explicit correlation between some proteins in urinary proteome and the development of diseases [8,9,10].

Biochemical profiling of clinical samples using attenuated total reflectance–Fourier transform infrared (ATR-FTIR) spectroscopy is an easy analytical technique that does not require sample preparation [11,12]. Different spectrum regions correlate with different biochemical moieties, and the investigation of such regions can potentially reveal altered biochemical molecule classes. More importantly, IR fingerprint regions can represent a unique snapshot of the complex biochemical profiles of clinical samples. Such fingerprints, when combined with non-linear machine leaning models, can identify discriminant features that are associated with health and disease [13,14]. Spectral profiling of biofluids such as blood, urine and saliva is an extremely promising source of liquid biopsy-based assays [15,16].

The ATR-FTIR spectroscopy-based liquid biopsy test, a vibrational spectroscopy techniques was used in a recent clinical trial on suspected brain cancer patients, alongside clinical assessment in primary care to help achieve earlier cancer detection and diagnosis [17]; this technique gives more accurate classification for early-stage tumors than other liquid biopsy approaches that are based on tumor genetic material such as circulating DNA [18]. Any changes in FTIR peaks could be a consequence of the induced significant alterations in molecular characteristics as well as arrangement, and structure and dynamics in tissues, membranes, and cells. These changes could help differentiate between diseased and non-diseased patients [12].

FTIR spectroscopy is a promising tool to diagnose a wide range of diseases such as Alzheimer’s [19], prostate cancer [20], bladder cancer [21], and viruses such as COVID-19 [22].

Therefore, ATR-FTIR spectroscopy has the potential to become a useful diagnostic tool that is deliverable and feasible in the healthcare system [23,24].

Here, we have examined the performance of a rapid spectroscopy-based liquid biopsy test in relation to tumor recurrence in non-muscle invasive bladder cancer patients. The spectral data coupled with machine learning algorithms have been used to differentiate between this recurrent cancer and non-recurrence (control) patients to identify an FTIR fingerprint for NMIBC recurrence.

## 2. Results

### 2.1. Overview FTIR Spectra Dataset

**Figure 1:**
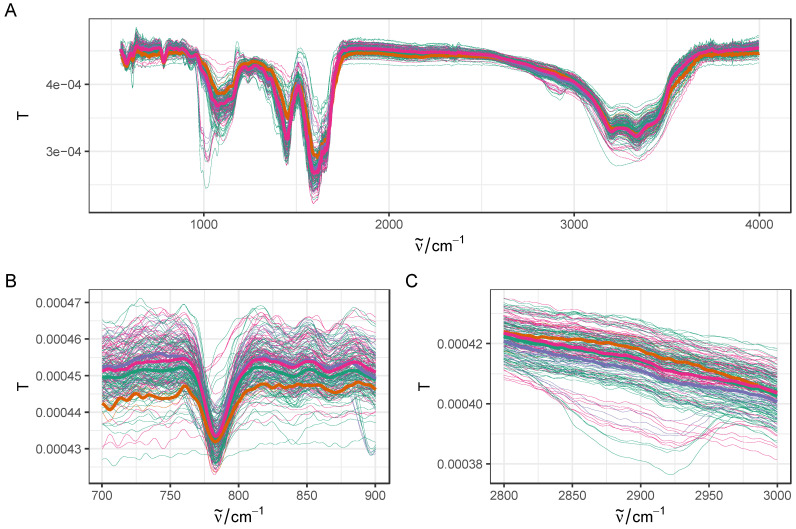
Overview of FTIR dataset (baseline corrected and vector normalized), with full range (**A**), fingerprint region (**B**), and hydrocarbon C-H stretching region (**C**). Bold lines represent average spectra in each group.

Urine samples from previously diagnosed NMIBC patients were collected during their first follow-up visit prior to cystoscopy examination. The pathological results of cystoscopy were recorded for a total of 62 individuals (Table 1). Since the aim of this study was to detect NMIBC recurrence, we further collapsed the patient samples into two groups, recurrence (*n* = 21) and NMIBC-free, which includes all other cystoscopy results (*n* = 41). Spectroscopic FTIR measurements were recorded at least in triplicate, and exported from Bruker software (Opus 6.5) after baseline correction, as described in the Methods section. Empty and corrupted spectra files were discarded, resulting in a total of 187 spectra that were included in the downstream analysis. For initial data exploration, raw spectra were first vector-normalized and different regions were visualized as shown in Figure 1.

### 2.2. Assessment of Technical Variability and Batch Effects

Spectra were acquired on seven different days, which may introduce technical variability batch effects, affecting the downstream analysis. Several studies and tools have investigated batch effects in FTIR, which can arise from atmospheric changes such as humidity and temperature, operator variability, or instrument calibration [13,25]. We addressed the batch effects by randomizing the acquisition with respect to the NMIBC status. Additionally, one of the samples was designated as a quality control (QC) sample and measured repeatedly in all batches.

**Figure 2:**
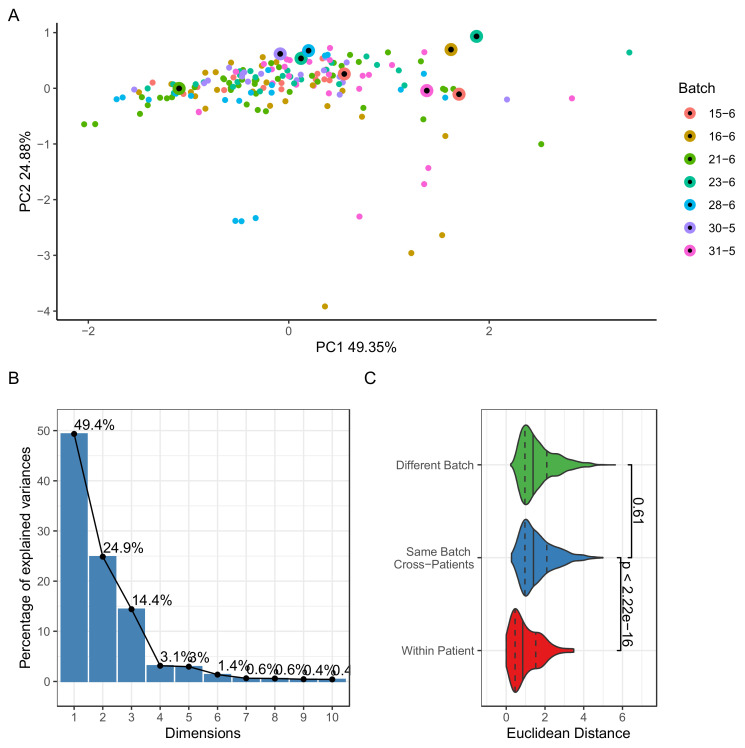
Assessment of technical variability and batch effects. (**A**) Principal component analysis showing the first two components colored by batch. Quality control samples are marked with black dots. (**B**) Scree plot showing variance explained by top 10 principal components. (**C**) Violin plots showing the distribution of Euclidean distances between spectra from the same patient, across patients and batches. The vertical lines represent the first, second, and third quartiles. Wilcoxon Rank sum *p* values are shown between groups.

We examined the PCA plots and colored the samples by batch (Figure 2A,B). The plots revealed a high variance and explained the first principal component (49.53%); however, they did not show any remarkable separation between samples from different batches. QC samples (Figure 2A, black dot markers) were not clustered with samples from several batches. Taken together, the results did not indicate any significant batch effects, despite the high variability within the dataset.

Next, we investigated whether the technical variability was less than the biological variability by comparing spectra acquired from replicates and across different samples and batches. The distribution of pairwise Euclidean distance was used to evaluate how close the spectra were. Figure 2C confirmed that the distances between the replicate spectra were significantly lower than for spectra from patients ((Wilcoxon-rank sum test, *p* < 2.22 × 10^−16^). We concluded that the technical variability was lower than the biological signal pursued in this analysis.

### 2.3. Preprocessing Parameters and Model Selection

To create a classifier for NMIBC status using FTIR spectra, we created a two-class response variable, *recurrence*, to denote NMIBC diagnosis by Cystoscopy (*n* = 21), and *free* to include all other diagnoses (*n* = 41). Using recurrence as the positive response class, we then assessed 35 different machine learning models from the *caret* R package. Since feature selection and spectral preprocessing can significantly alter the performance of the assessed models, we sought optimal processing parameters for each model. Through a hyperparameter grid search, we evaluated 25,200 combinations, optimizing spectrum range, normalization, binning, [26] filter derivative, and window size (Table 2). Notably, the selected four spectrum ranges correspond to biological regions of interest, full spectrum 500~4000 cm^−1^, bacterial fingerprint region 700~900 cm^−1^, extended fingerprint region 700~1800 cm^−1^, and hydrocarbon C-H stretching region 2800~3000 cm^−1^.

**Figure 3:**
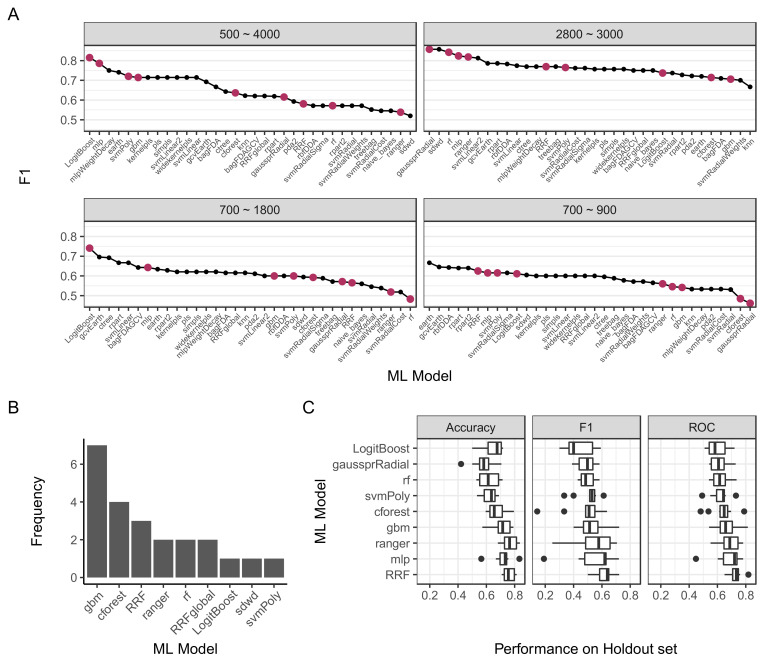
Machine learning model selection and validation. (**A**) Top performance F1 score for each model against different spectrum ranges. Maroon dots represent models selected for further validations. (**B**) Models with high accuracy in both training set, as estimated by ROC > 0.8 in cross-validation, and in the test set (F1 score > 0.5). Frequency indicates the number of processing parameter combinations in a specific model achieved by these criteria. (**C**) Performance scores of selected models on test sets. Dataset splitting and model fitting were repeated 10 times, and performance was evaluated with three metrics.

Different numbers of patients in the NMIBC-free (*n* = 41) and recurrence (*n* = 21) groups created a class imbalance that can affect model training. We addressed the class imbalance using two techniques. First, we used ROC as a performance metric to optimize the classifier models. Second, we utilized the synthetic minority over-sampling technique (SMOTE) [27] to impute new data points for the minority class, recurrence in our case. The preprocessing combinations for each model were assessed on the holdout test set and the top performing combination was plotted in Figure 3A. Based on performance on the test set, we selected five models for further validation, *gaussprRadial*, *rf*, *LogitBoost*, *mlp*, and *svmPoly*.

Because of the large number of combinations that were evaluated relative to the dataset size, dataset splitting into training and test sets may incorrectly inflate performance in the holdout set. In such cases, high test set performance correlated with a poor training set performance, which indicates poor model robustness (Table S1). To address this issue, we introduced two additional criteria: (1) requiring good performance in both training and test set, and (2) the model should give similar high performance in similar preprocessing conditions. Consequently, we filtered models with training ROC > 0.8 and F1 test performance > 0.5, and selected four additional models that frequently matched these criteria (Figure 3B), *gbm*, *cforest*, *RRF*, and *ranger*. The final list of selected models and processing parameters is shown in Table 3.

### 2.4. Model Validation and Tuning

**Figure 4:**
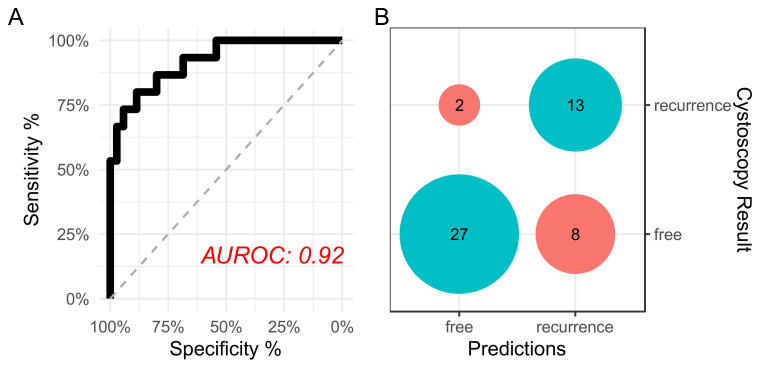
Final model performance. (**A**) Receiver operating characteristic curve showing the area under the curve. The curve was constructed using class probabilities obtained from the final RRF model against the holdout test set; (**B**) confusion matrix of the performance of the binary classifier.

To assess the robustness of the model against dataset splitting, we repeated model training and testing 10 times for each of the selected models, where we reconstructed different training and test sets for each iteration by setting different random number generator seeds. We subsequently assessed the performance of the models using three metrics, accuracy, F1, and AUROC. Based on the results shown in Figure 3C, we concluded that the regularized random forest (RRF) model was the best-performing model. To investigate RRF model tuning, we investigated the constructed models (Table S2), which indicated 2 as the optimal number of randomly selected predictors, a regularization value of 1.000, and an importance coefficient of 0.0. The final selected model achieved an AUROC of 92% with 86% sensitivity and 77% specificity (Figure 4).

### 2.5. Extraction of Feature Importance

**Figure 5:**
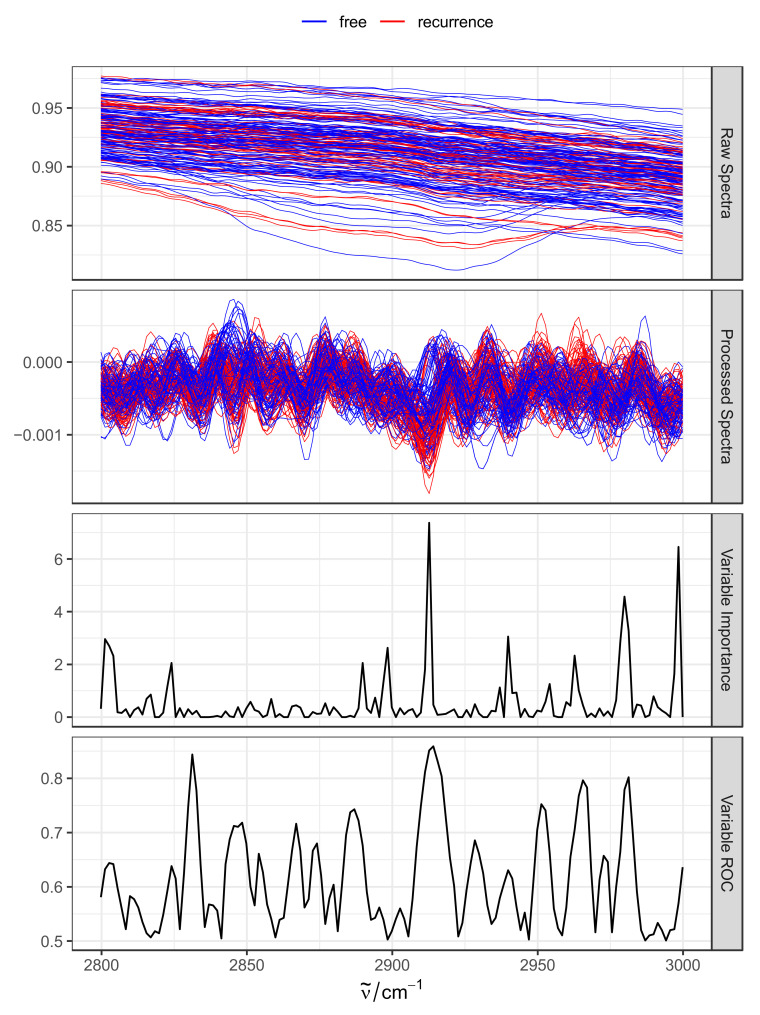
Investigation of wavenumber ranges contributing to model performance. Raw and processed spectra for the selected spectrum range (top two panels) are shown, colored by NMIBC status. Variable importance scores (third panel) are directly obtained from the model using out-of-bag estimation strategy. Variable ROC scores are obtained via similar strategy but evaluating estimates against the test set.

Random forest models enable estimating variable importance by shuffling predictor values and measuring the performance using out-of-bag samples [28,29]. This technique not only allows for the calculation of per-feature importance scores, but also ROC estimates. We exploited this feature to calculate importance and ROC estimates, and to visualize them alongside spectra. As shown in Figure 5, variable importance and predictive ROC spikes around 2912 cm^−1^ were characteristic of lipid CH_2_ asymmetrical stretching. An additional spike is also observed around 2980 cm^−1^, reflecting the stretching vibrations of methyl hydrocarbon chains. An investigation of processed spectra (Figure 5, second top panel) clearly reveals spectral differences between recurrence (red) and NMIBC-free (blue) samples. Taken together, the variable importance results suggest an altered lipid profile in urine in recurrent NMIBC.

## 3. Discussion

Non-muscle invasive bladder cancer (NMIBC) is low-grade urothelial carcinoma that is commonly treated by transurethral resection (TURBT). While not life threating, the high risk of recurrence and progression for muscle-invasive bladder cancer necessitates regular patient follow-ups to detect and prevent progression. However, the gold standard for NMIBC detection still relies solely on cystoscopy, an invasive procedure and source of major discomfort for patients.

It has been estimated that urine markers would need to have a sensitivity between 90–95% to replace cystoscopy [30,31]. Several cytological assays have been reported previously, with sensitivities ranging from 7–93% [32]. Other multivariate biomarkers have been suggested based on gene expression studies [33,34]; however, none were able to achieve clinical utility [4].

In this study, we attempted to establish a rapid, easy-to-administer, and non-invasive assay for the detection of NMIBC in follow-up patients. The simple assay relies on FTIR spectroscopic measurement of urine samples with no sample preparation required. Using cystoscopy examination and pathological investigation of biopsies as the ground truth, we used machine learning to establish an accurate classifier from acquired spectra. The achieved sensitivity was 86%. Although our study did not reach the sensitivity sought after for clinical application, this is a pilot study and performance can be strongly affected by outliers and inter-individual variations. Nevertheless, these promising results present a great potential for improvement with a larger cohort size.

Exploring the machine learning models revealed a strong predictor signal in the spectrum range 2800~3000 cm^−1^, a characteristic region for stretching vibrations of lipid hydrocarbon chains. Altered lipid metabolism has previously been generally implicated in cancer [35,36], and specifically in NMIBC [37]. Spectroscopic evidence of changes in the serum levels of biochemical molecules including lipids has also been reported [38].

To the best of our knowledge, this study is the first report on this change in the FTIR spectrum that ranges from 2800 to 3000 cm^−1^. Because of this explicit fingerprint, this spectroscopic technique can be used as a diagnostic tool for the early detection of recurrent NMIBC. Unlike other tools and traditional techniques in the literature that have used complex procedures, our study proposed a direct and simple approach to detect NMIBC patients’ recurrence. Interestingly, only a few studies have been published addressing bladder cancer in terms of using fluids, such as urine and bladder washing water. When we compared and contrasted these studies to our FTIR technique, we found that only one of them dealt with bladder wash [21] and solely for the bladder cancer detection marker, not recurrent NMIBC. In contrast, their fingerprint FTIR area had a different specific range than what we have found here. Gok et al. listed FTIR with a broad range from 1500 to 800 cm^−1^ as a fingerprinting tool for bladder cancer in general, where spectroscopic evidence of changes in serum levels of biochemical molecules, including lipids, has also been reported.

## 4. Materials and Methods

### 4.1. Patients and Samples Collection

A total of 62 patients were recruited after Transurethral Resection of Bladder Tumor (TURBT) of non-muscle invasive bladder cancer (NMIBC) and included in this prospective study. All patients were subjected to diagnosis and treatment at the National Cancer Institute (NCI), Cairo, Egypt.

Urine samples for cytology were obtained and abdominal ultrasound and cystoscopy were performed at three-month-intervals after Transurethral Resection of Bladder Tumor (TURBT) according to European Association of Urology (EAU) guidelines [4]. Cystoscopy was considered as the standard method for recurrences diagnosis. In the case of positive urine cytology, CT program and random bladder biopsies were performed. The voided urine specimens were divided into two aliquots; one was prepared for cytopathological examination and the other was stored at −80 °C for the FTIR assay.

The study was conducted in accordance with the Declaration of Helsinki, and the protocol was approved by the Ethics Committee of the National Cancer Institute (NCI), approval number (2111-502-015). All participants provided a written informed consent which was signed by each patient.

### 4.2. ATR-FTIR Measurement

A BRUKER ALPHA FT-IR spectrometer, equipped with a ZnSe crystal attenuated total refraction (ATR) accessory, was used for recording the infrared spectra. A deuterated triglycine sulfate (DTGS) detector was used for measurements. The background interferogram was recorded with a clean ZnSe surface. After shaking the urine container, 5 µL of the sample was pipetted onto the ZnSe crystal surface. Water absorption hinders the appearance of many components in the spectrum; therefore, the sample was dried for 15 min using a gentle stream of N_2_ gas prior to data acquisition to remove excess water. All samples ware measured by collecting and averaging 28 scans for a final resolution of 4 cm^−1^.

### 4.3. Spectral Data Pre-Processing

Each urine sample was loaded and acquired at least three times. Spectral data were baseline corrected using vendor software and exported as SPC files. The files were then imported into R statistical environment (v4.2) for analysis using hyperSpec package v0.100.0 [39]. Spectral derivative transformations and Savitzky–Golay filtering [26] were performed using Signal R package (v0.7-7) [40], with a polynomial order of 3 and a window size ranging from 5 to 13. Raw, first or second order derivative transformations were performed as part of hyperparameter optimization. Transformed spectra were either not normalized, normalized to Amide-I band maximum in the 1500–1700 cm^−1^ range, or to Urea band maximum in the 1400–1500 cm^−1^. Outliers were identified and removed through the *pcout* method from *mvoutlier* R package (v2.1.1). Wavenumber ranges 700–900 cm^−1^, 700–1800 cm^−1^, and 2800–3000 cm^−1^ were extracted where relevant.

### 4.4. Machine Learning

We relied on the extensive functionality implemented in caret R package (v6.0-92) to perform dataset partitioning, model training, and testing. A comprehensive guide on the implemented models and configurable parameters is available in Kuhn [41]. Following outlier removal, processed spectra were randomly split into training and testing sets in a 70:30 ratio. Replicate spectra from the sample urine samples were kept together to prevent data leaking. Thirty-five machine learning models were evaluated as detailed in Table S3. Two-class (free vs recurrence) variable was used for training and prediction. Model training was performed on the dataset using a 10-fold repeated cross-validation. To account for replicate spectra, we used a stratified K-fold sampling strategy, ensuring that replicates were always contained together in single fold. To address the class imbalance, ROC measure was used as a cost function instead of accuracy. Additionally, synthetic minority over-sampling technique (SMOTE) [27] was used to create new data points for the minority class. Trained models were then evaluated on the holdout testing set using F1 and ROC scores where appropriate.

## 5. Conclusions

This study presented a rapid and easy spectroscopic assay to detect NMIBC recurrence from urine using FTIR and sophisticated machine learning modeling. We examined the performance of 35 machine learning models using a training set (70%) and a held-out test set (30%). With 86% sensitivity and 77% specificity, a regularized random forest model achieved an area under the receiver operating characteristic (AUROC) of 0.92. This technology can be further utilized to describe the transition to the defined application of ATR-FTIR spectroscopy using urine samples for the detection of recurrence in NMIBC patients and the subsequent impact on clinical sectors. This can be done by simply using the spectroscopy of urine instead of undergoing the complications associated with using cystoscopy, which is usually uncomfortable and sometimes painful. Therefore, this technological advancement leads to a prospective clinical validation study that is conducted in the population that is the focus of this article, which presents exploratory findings that confirm the fingerprint correlation with NMIBC. In addition, this finding can be used as an alternative diagnostic tool to detect NMIB cancer at an earlier stage, or it might completely replace cystoscopy with the use of a urine test in the clinic. This allows for early intervention and appears as a step forward in both the advancement of technology, and the improvement of the clinical course of treatment for patients who suffer from this cancer or in the diagnosis of this disease. We hope to further improve the current assay in a larger cohort to assess its potential in NMIBC surveillance.

## Figures and Tables

**Table 1:** Cystoscopy results for the cohort.

Cystoscopy Result	Number of Patients
Free	31
Hyperplasia	1
Inflammation	9
Recurrence	21

**Table 2:** Preprocessing parameters for spectra.

Preprocessing Parameter	Variants
Spectrum Range	500~4000 cm^−1^
	700~900 cm^−1^
	700~1800 cm^−1^
	2800~3000 cm^−1^
Savitzky-Golay Derivative	0, 1, 2
Normalization	No normalization
	Amide 1500~1700 cm^−1^
	Urea 1400~1500 cm^−1^
Savitzky-Golay Window Size	5, 7, 9, 13
Bin size	1, 2, 3, 5, 10

**Table 3:** Models selected for further validation with their processing parameters.

Model	CV ROC	Test Accuracy	F1 Score	Spectrumrange	SG Derivative	SG Window	Bin Size	Normalization Peak
cforest	0.83	0.74	0.62	2800~3000	1	13	1	urea
gbm	0.81	0.79	0.67	2800~3000	1	7	1	none
ranger	0.82	0.77	0.64	2800~3000	1	13	1	urea
RRF	0.82	0.81	0.71	2800~3000	1	13	1	urea
gaussprRadial	0.46	0.92	0.86	2800~3000	0	9	1	none
LogitBoost	0.53	0.88	0.81	500~4000	1	9	3	amide
mlp	0.67	0.85	0.82	2800~3000	0	7	2	amide
rf	0.42	0.92	0.84	2800~3000	0	5	2	none
svmPoly	0.7	0.85	0.76	2800~3000	2	7	10	amide

CV: cross-validation; SG: Savitzky-Golay filter; model abbreviations are provided in Table S3.

## Data Availability

Raw and processed FTIR spectra are available upon request.

## Supplementary Materials

The following supporting information can be downloaded at: https://www.mdpi.com/article/10.3390/molecules27248890/s1, Table S1: Performance of hyperparameter combinations, Table S2: Model tuning of regularized random forests, Table S3: Machine learning models abbreviations.
